# A Retrospective Analysis of the Underlying Health Status of Patients Treated for Stroke in the Emergency Department of a Community Hospital Situated in a Health Professional Shortage Area

**DOI:** 10.7759/cureus.68150

**Published:** 2024-08-29

**Authors:** Evelyn B Voura, Tabatha M Jorgensen, John R Stulb, Margaret E Mulligan, David J Padalino

**Affiliations:** 1 Crouse Neuroscience Institute, Crouse Health at Crouse Hospital, Crouse Medical Practice, PLLC, Syracuse, USA; 2 Neuroscience and Physiology Department, State University of New York Upstate Medical University, Syracuse, USA; 3 Math and Science Division, Dominican University New York, Orangeburg, USA

**Keywords:** primary care, smoking, hyperlipidemia, hypertension, diabetes, health professional shortage area, health disparities, medically underserved, medical determinants of health, stroke

## Abstract

Background

Hypertension, diabetes, and hyperlipidemia are known contributors to the incidence of stroke. These and other risk factors such as smoking can be managed with effective primary care, but living in a medically underserved area and racial background can limit access, thereby deleteriously affecting underlying medical conditions and disproportionately contributing to negative stroke outcomes. Our goal is to learn about the on-admission health of 1,731 stroke patients who presented to the Crouse Hospital emergency department (ED) between January 2019 and January 2021 to better understand the circumstances affecting these patients. Crouse Hospital is a community hospital in Syracuse, New York, and an award-winning comprehensive stroke center in the region. The hospital is located in a health professional shortage area (HPSA) and serves both rural and urban patients of various ethnic backgrounds and socioeconomic statuses.

Methodology

We retrospectively examined the stroke patient data to determine how access to primary care and race affected smoking status, arrival time following the onset of symptoms, stroke severity, thrombolytic administration, and metrics relating to hypertension, diabetes, hyperlipidemia, and depression.

Results

We determined that, while most patients stated that they had a primary care provider, stroke incidents were typically associated with high blood pressure and high blood glucose despite the prevalence of prescriptions to treat these conditions and that both conditions affected the underserved and non-White patients (Black, Hispanic, Asian, Indigenous, and Other) more so than the served and White populations. Underserved and non-White patients, were also more likely to be associated with smoking behavior.

Conclusions

The data indicated the major health factors affecting the patients and highlighted those influenced by limited access to primary care and racial background. As a result, we developed a survey to gauge patients' perspectives on primary care and underlying medical conditions before and after their stroke. This patient-centered approach will help refine our stroke education efforts to improve stroke outcomes in the community.

## Introduction

Stroke is a leading cause of death and serious long-term disability globally, costing an estimated US$ 891 billion [1,2]. In the United States, stroke is a leading cause of death and disability, with the risk being higher for those who are not White [3,4]. Accordingly, the Centers for Disease Control and Prevention (CDC) published recommendations on how best to avoid stroke, highlighting “living healthy” and working with healthcare providers to manage key medical conditions - heart disease, diabetes, and high blood cholesterol - along with taking prescribed medications, smoking cessation, reducing alcohol consumption, and maintaining a healthy diet [5]. Healthcare providers are also mandated to provide education on how to manage stroke risk factors and improve post-stroke outcomes [6,7]. Despite these efforts, patients continue to exhibit poorly controlled chronic health conditions at the time of their stroke [2,8].

Hypertension is considered the most salient modifiable risk factor since it can be treated using both medication and lifestyle changes [9-11]. Diabetes is an additional health concern because it can double the chances of stroke and has an added detrimental effect on stroke outcomes. Diabetes is also a prime target among modifiable risk factors because it affects many younger stroke patients [12]. The contribution of hyperlipidemia to stroke is study-dependent and is comparably underemphasized in the literature. It is recognized, however, that high cholesterol levels lead to poorer stroke outcomes and that statins reduce the risk of ischemic stroke [9,12].

Limitations on accessing healthcare and a poor understanding of stroke are also important contributing factors, leading to the higher burden from and increasing rate of stroke among younger individuals and those with less education [13-15]. Low socioeconomic status and being from a racialized group further augments stroke risk from hypertension, diabetes, and high blood lipids, with these issues being further compounded in low- and middle-income countries with resource-strapped healthcare systems [2,3,16-18].

In the United States, fundamental to the increasing risk of stroke among marginalized groups is diabetes - particularly among the Hispanic population, while hypertension is also highlighted within the Black community [9,16,19]. Therefore, according to the study by Howard et al. [20], people who are Black, when compared to those who are White, have a higher rate of first stroke at a younger age. Since the rate of stroke is projected to increase overall, and disproportionately so among rural, younger, and racialized individuals, it is important to take action to reduce the effect of underlying medical conditions with an emphasis on these groups [3,21,22].

While the goal of addressing the underlying reasons for stroke in the population may seem straightforward, in reality, those factors vary considerably at the individual level [9]. A number of assessment tools have been developed to help primary care providers (PCPs) work with patients to understand their likelihood of having a stroke [2,23]. Unfortunately, the practical use of these resources has proven inconsistent at the provider level, and the assessments lack racial and cultural sensitivity, leading to unreliable results [24,25]. Adding to the complexity of providing a clear perspective of personal stroke risk to patients is that patients can suffer from multiple compounding comorbidities [11,26].

One in three Americans is affected by at least one stroke risk factor, and statistics indicate that diabetes in particular not only doubles the risk of stroke but also increases the rate of cardiovascular disease and depression [4,27-29]. Even prediabetes is associated with an increased risk of stroke [30]. Published reports stress both hypertension and diabetes as the prime health factors to monitor in terms of stroke risk [16,31]. The understanding that patients typically suffer from multiple co-determinants prior to their stroke led to the development of strategies integrating care to support patients in addressing lifestyle changes focused on weight management, physical activity, diet, smoking, and alcohol consumption [32].

Despite our understanding of the factors leading to stroke and years of effort to educate the public, the number of recurrent strokes has held steady for the past decade, indicating that efforts to mitigate the impacts of hypertension, diabetes, and hyperlipidemia, as well as other modifiable risk factors, has not been successful - with patients demonstrating that they do not appreciate their personal stroke risk [2,33]. Taking a grassroots approach to educate communities about stroke, however, was shown to increase stroke symptom awareness, and a patient-centered approach could help modify behaviors [34,35].

Personalized care will be important to make a meaningful change in the incidence of stroke overall, particularly among marginalized groups and those with poor access to primary care [2,16,32,36,37]. There is an ongoing need to help individuals get their stroke risk factors under control, but the ability of a patient to do so can be limited by their understanding of medical concepts and their access to consistent primary care [38-40]. Our objective was to understand the on-admission health status of individuals who came to the Crouse Hospital emergency department (ED) for stroke and use that knowledge to develop a plan to help improve stroke outcomes in the community.

## Materials and methods

Patient population

This retrospective analysis of patient information began in February of 2021 using 1,731 stroke incidents occurring between January 2019 and January 2021. We extracted the data from ED records, as well as laboratory analyses and health professional notes taken from hospital admission until the time of discharge. The dataset was selected as a subset of charts of all patients having a diagnosis of stroke (ischemic and hemorrhagic) or transient ischemic attack (TIA) from the Crouse Hospital ED during that time. A starting group of 1,863 incidents (as of February 24, 2021) was reduced to those that were associated with home addresses in the six counties surrounding Crouse Hospital (Onondaga, Oneida, Oswego, Madison, Cayuga, and Cortland). The individuals involved in data extraction had access to patient-identifying information during data collection; however, all identifiers were removed prior to all data analysis. The patients were grouped as served or underserved and White or non-White patients (i.e., Black, Hispanic, Asian, Indigenous, and Other). As with our previous publication, we assessed service status (served or underserved) using the measurement tool provided by the United States Health Resources and Services Administration (HRSA), referred to as a health professional service area (HPSA), which assigns a score based on the home address of the patient [41]. The scoring method involves the tabulation of multiple metrics, including the population-to-provider ratio, the percent of the population in the area below the 100% federal poverty level, the infant health index (involving the infant mortality rate or low birth weight rate), and the travel time to the nearest source of care outside the HRSA designation area. A score on the HRSA scale indicates that the area is considered a priority for physician placement and additional Medicare reimbursements - a HPSA (an underserved area for our study).

Medical factors

Building on the foundation of our previous study examining how social determinants were related to the stroke outcomes of the patients, here, we assessed how access to primary healthcare services related to the underlying medical condition of patients arriving at the hospital ED suffering from a stroke [42]. We assessed the metrics with a focus on service level (served and underserved), as well as race - in this case, White or non-White patients, as described above. The medical factors used for comparison between these groups were categorized and assessed as background data, hypertension, diabetes, lipids, and depression. The details for each of these categories are described below.

Background Data

To understand the background of the stroke presentation and history of the patients, we assessed the type of stroke (ischemic, hemorrhagic, or TIA), as given by the final billing codes for each patient, their last known well (LKW), the results of their National Institutes of Health Stroke Score (NIHSS) determination, their smoking status, and if they were seen previously in the ED for a stroke or TIA. The LKW (when they were last at their baseline or when they last felt normal) was categorized as less than three hours, three to six hours, six to 12 hours, 12-24 hours, and greater than 24 hours. We further assessed the second class (three to six hours), for those patients who arrived less than 4.5 hours from their LKW. These patients were then grouped with those who arrived three hours from their LKW. This extra LKW analysis (less than 4.5 hours, including all of the patients in the less than three-hour group and a subset of the three to six-hour group) allowed us to determine which ischemic stroke patients arrived in the ED within a time window that would allow for the use of a thrombolytic (tissue plasminogen activator; tPA in this case) for ischemic stroke recovery. We then assessed the proportion of those patients with an ischemic stroke and an LKW of less than 4.5 hours for those who received tPA. Importantly, in addition to the type of stroke and LKW, NIHSS analysis and imaging data are used to determine the type of immediate intervention necessary (such as the use of tPA) and assess the post-acute care disposition of the patient [43]. With a score of 0 considered normal, the higher the NIHSS score the worse the neurological deficit of the patient (the highest possible score being 42). We examined the patient's history for evidence of a previous stroke or TIA and recorded this information. Our assessment of smoking status included if the patient was currently a smoker (Yes), never smoked (No), or quit smoking at some point (Former).

Hypertension

Hypertension was assessed by focusing on the systolic extraction reading of the first available blood pressure taken for each patient. We studied the average systolic extraction for the data and then categorized the pressures as low (< 90 mmHg), normal (≥ 90 mmHg and < 120 mmHg), elevated (≥ 120 mmHg and < 140 mmHg), high stage 1 (≥ 140 mmHg and < 160 mmHg), high stage 2 (≥ 160 mmHg and < 180 mmHg), and crisis (≥ 180 mmHg). We further analyzed the data by looking for laboratory results for troponin, and these were grouped as normal (< 0.05 ng/mL) or high (≥ 0.05 ng/mL). Note that, for a subset of the data collected in 2019, a troponin value < 0.06 ng/mL was considered normal; however, we opted for the current hospital standard in the data analysis as indicated (< 0.05 ng/mL). We also assessed the study population by looking at the home medications list for aspirin and drugs used for the treatment of high blood pressure.

Diabetes

Several methods were used to assess for diabetes among the patients. The blood glucose concentration from the first laboratory report was recorded and studied. We compared the mean glucose concentration between the patient groups and also categorized the measurements as high (≥ 100 mg/dL) or normal (< 100 mg/dL). Additionally, we assessed the glycated hemoglobin/HbA1c (A1c) levels of the records that included this analysis and again classified the value as high (> 6%) or normal (≤ 6%). We also examined the home medication list of each patient for drugs used to treat diabetes. When analyzing these, we did not distinguish between insulin or non-insulin medications.

Lipids

To assess for hyperlipidemia, the mean blood low-density lipoprotein (LDL) concentration was used as the main measurement. We compared the mean LDL concentration of each group and assessed the values overall as high (> 100 mg/dL) or normal (≤ 100 mg/dL). The other metric that was used for the lipids analysis was whether or not each record included medications to treat high blood lipids in the home medication list for the patient. Here, we did not distinguish between statins or other lipid-lowering treatments.

Depression

The data used to assess depression was the inclusion of medications to treat depression in the home medications list.

Statistical analysis

We determined the significance of the metrics using separate multivariate analyses for individuals living in underserved areas (HPSAs) compared with those living in served areas and then repeated these assessments by examining the differences between White patients and those who were not White. We determined the significance of these associations using an analysis of variance (ANOVA) and t-tests (two-tailed where applicable) for continuous variables and Pearson chi-square tests for categorical variables. The data were considered significant, with a p-value of less than 0.05. For charts where the data on a particular data point was not available, those were considered as “No” for the Pearson chi-square tests to keep the number of incidents the same for served, underserved, White, non-White, and combined categories. This allowed for the direct comparison of the different metrics between the various medical aspects being assessed. For mean values assessed using ANOVA, the charts missing data were removed from the calculation. Symbols were used to indicate significance at the following levels using an analysis of variance of independent t-test/ANOVA for continuous variables and Pearson chi-square tests for categorical variables: †p ≤ 0.10, *p ≤ 0.05, **p ≤ 0.01, and ***p ≤ 0.001. The statistical analyses were conducted using either SAS (version 9.4) or Microsoft Excel (Office Professional 2019, Microsoft® Corp., Redmond, WA).

Ethics statement

Data collection and procedures for this study were approved by the Crouse Hospital Institutional Review Board. Since this study involved a retrospective review of patient data and did not pose a risk to the health of the patients, informed consent did not apply. To protect the personal information of the patients, the data file was stripped of identifiers prior to analysis.

## Results

Background data

**Figure 1:**
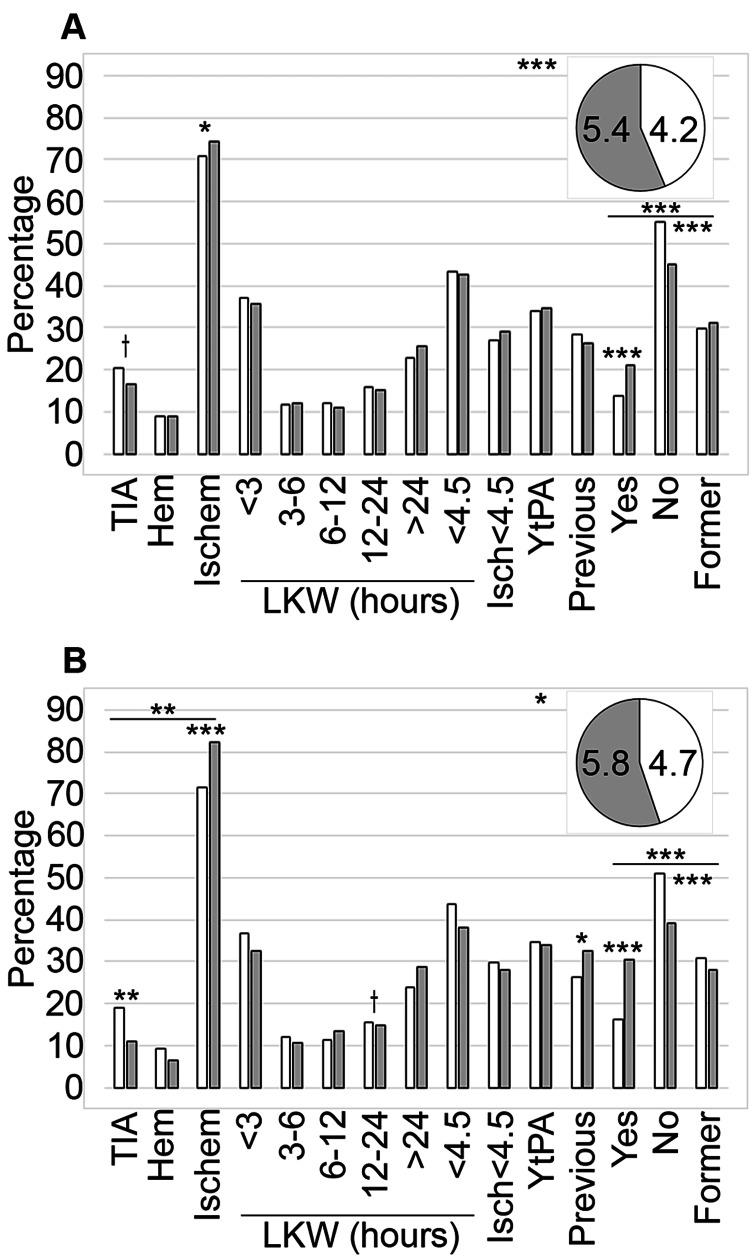
Comparative analysis of background metrics. Data focused on understanding the stroke background and smoking status were compared between served and underserved (A) and White and non-White (B) patients. Served patients (n = 712) are represented by the white bars and a pie piece, and underserved patients (n = 1,019) by the grey bars and a pie piece in (A). Meanwhile, White patients (n = 1,514) are indicated by the white bars and a pie piece, and non-White patients (n = 217) are shown using the grey bars and a pie piece in (B). The mean NIHSS values are shown in the pie chart inset. TIA: % coding for transient ischemic attack; Hem: % coding for hemorrhagic stroke; Ischem: % coding for ischemic stroke; Isch<4.5: % of stokes that were ischemic with a LKW less than 4.5 hours; YtPA: % of ischemic strokes with a LKW less than 4.5 hours that received tPA; Previous: % of patients with a previous stroke on record Yes, No, and Former refer to the % of patients in each of these smoking categories. For the YtPA proportions, the number of patients (n) with ischemic strokes arriving less than 4.5 hours from their last known well used for the calculations were 192 for the served, 296 from the underserved, 423 from the White category, and 65 from the non-White category. Symbols indicate significance at the following levels: †p ≤ 0.10, *p ≤ 0.05, **p ≤ 0.01, ***p ≤ 0.001.

The patients were mostly White (87.5%). The non-White category included patients identifying as Black (9.9%), Hispanic (1.3%), Asian (0.5%), Indigenous (0.1%), or Other (0.8%) [42]. We expanded our assessment of the NIHSS results and the use of tPA on this dataset here to include a comparison with all racialized patients and, for the tPA results, provided a further analysis comparing the use of tPA with medical considerations, including the LKW and stroke diagnosis [42]. We observed significant differences between the mean NIHSS data for the served (n = 679) and underserved patients (n = 970; p < 0.001; Table 1 and Figure 1A), as well as between the White (n = 1,437) and non-White populations (n = 212; p = 0.031; Table 2 and Figure 1B).

**Table 1:** Analysis of background data for the served and underserved populations. †p ≤ 0.10, *p ≤ 0.05, **p ≤ 0.01, ***p ≤ 0.001

	Served (n = 712)	Underserved (n = 1,019)	p-value
NIHSS (mean)	4.2	5.4	0.000***
Stroke Classification (%)			0.158
TIA	20.22	16.68	0.060†
Hemorrhagic	6.99	8.83	0.910
Ischemic	70.79	74.48	0.044*
Last Known Well (LKW, %)			0.839
< 3 hours	37.22	35.72	0.524
3 to 6 hours	11.66	11.87	0.890
6 to 12 hours	12.08	11.09	0.525
12 to 24 hours	15.73	15.21	0.769
> 24 hours	22.89	25.71	0.180
< 4.5 hours (for tPA)	43.40	42.49	0.708
Ischemic and LKW < 4.5 hours (% of underserved)	26.97	29.04	0.344
% of ischemic and LKW < 4.5 hours to receive tPA	33.85	34.80	0.830
Evidence of previous stroke (%)	28.37	26.20	0.318
Smoking Status (%)			0.000***
Yes	13.76	21.20	0.000***
No	55.34	45.24	0.000***
Former	29.63	31.01	0.540

**Table 2:** Analysis of background data for the White and non-White populations. †p ≤ 0.10, *p ≤ 0.05, **p ≤ 0.01, ***p ≤ 0.001

	White (n = 1,514)	Non-White (n = 217)	p-value
NIHSS (mean)	4.7	5.8	0.031*
Stroke Classification (%)			0.003**
TIA	19.15	11.06	0.004**
Hemorrhagic	9.25	6.45	0.176
Ischemic	71.6	82.49	0.000***
Last Known Well (LKW, %)			0.460
< 3 hours	36.86	32.72	0.236
3 to 6 hours	11.96	10.60	0.562
6 to 12 hours	11.23	13.36	0.356
12 to 24 hours	15.52	14.75	0.087†
> 24 hours	23.98	28.57	0.141
< 4.5 hours (for tPA)	43.53	38.25	0.142
Ischemic and LKW < 4.5 hours (% of non-White)	29.95	28.14	0.537
% of ischemic and LKW < 4.5 hours to receive tPA	34.52	33.84	0.916
Evidence of previous stroke (%)	26.29	32.72	0.046*
Smoking Status (%)			0.000***
Yes	16.38	30.41	0.000***
No	50.86	39.17	0.000***
Former	30.78	28.11	0.424

Type of Stroke

Relying on billing codes to identify the type of stroke, we determined that, of the 1,731 incidents, 314 were diagnosed as a TIA (18.14%), 154 were hemorrhagic strokes (8.9%), and 1,263 were ischemic strokes (72.96%). Assessing the type of stroke by service category (Table 1 and Figure 1A), we did not observe a significant difference between the groups overall but did determine that the underserved (n = 1,019) had significantly more instances of ischemic stroke (p = 0.044; Table 1 and Figure 1A). Examining billing codes for type of stroke by race (Table 2 and Figure 1B), we determined that the overall variation of the type of stroke was significant (p = 0.003) and that non-White patients (n = 217) had more ischemic strokes (p < 0.001) and fewer instances of a TIA (p = 0.004) when compared to White patients (n = 1,514).

Last Known Well

The analysis of the LKW data indicated that there was no significant variation for the overall data or within time categories by service (Table 1 and Figure 1A) or by race (Table 2 and Figure 1B). LKW data were not available for three served patients (0.42%) and four underserved patients (0.39%) and was not available for seven (0.46%) of the White patients, but all the non-White patients had their LKW recorded.

TPA Administration

Our analyses did not indicate significant differences between the datasets for ischemic stroke with a LKW < 4.5 hours or for ischemic stroke with a LKW < 4.5 hours with tPA infusion by service (Table 1 and Figure 1A) or racial background (Table 2 and Figure 1B).

Prior Stroke

We examined each chart for evidence of a prior stroke or incidence of TIA and determined that there was no difference between the served and underserved (Table 1 and Figure 1A). Separating the patients by race on the other hand did result in a statistically significant variation between White (n = 1,514) and non-White (n = 217) patients (p = 0.046; Table 2 and Figure 1B).

Smoking Status

Examining the full set of patients we determined that 18.14% were classified as currently smoking, 49.34% stated that they were former smokers, and 30.44 % stated that they had never smoked. We did not have the information for 2.02% of the dataset. We observed a significant variation in smoking status overall by both service (p < 0.001; Table 1 and Figure 1A) and racial background (p < 0.001; Table 2 and Figure 1B). By smoking classification, significantly more of the underserved (n = 1,019) stated that they were active smokers (p < 0.001), and more of the served (n = 712) had never smoked (p < 0.001). By race, more non-White patients (n = 217) were active smokers (p < 0.001), while significantly more White patients (n = 1,514) stated that they never smoked (p < 0.001). Smoking status was not collected on 1.26% (n = 9) of the served and 2.55% (n = 26) of the underserved, respectively. Among White patients, 30 (1.98%) did not have their smoking status listed, while 2.3% (n = 5) of non-White patients did not have this information included in their chart data. Examining the proportion of smokers by HPSA status and ethnicity (Table 3), we observed a significant variation (p < 0.001) with the underserved non-White patients (n = 181) having the highest proportion of current smokers, while the served White population (n = 646) had the fewest patients in this category (Table 3).

**Table 3:** Analysis of the selected criteria referencing both service status and race. The combined high grouping for the hypertension categories allowed for Pearson chi-square analysis by grouping together the high stage 1, high stage 2, and crisis categories. Superscripts show the p-values for the two-tailed T-test analysis used to determine which of the values varied significantly as a source of variance in the overall ANOVA for the category. †p ≤ 0.10, *p ≤ 0.05, **p ≤ 0.01, ***p ≤ 0.001

	Served White (n = 676) patients	Served non-White (n = 36) patients	Underserved White (n = 838) patients	Underserved non-White (n = 181) patients	p-value
Current Smoker (%)	13.31	22.22	18.85	32.04	0.000***
Mean Systolic Pressure (mmHg)	149.5	151.6	149.4 ^t*(0.011)^	154.8 ^t*(0.011)^	0.060†
Hypertension Home Meds. (%)	73.07	66.70	69.45	74.59	0.546
Hypertension Categories (%)					
High stage 1	30.50	36.11	31.62	32.60	0.597
High stage 2	27.09	11.11	20.29	23.76	0.023*
Crisis	10.99	19.44	12.17	16.02	0.109
Combined high	68.58	66.66	64.08	72.38	0.207
Mean blood glucose (mg/dL)	131.25 ^t*(0.015)^^t**(0.001)^	147.28	139.73 ^t*(0.015)^ ^t*(0.030)^	157.34 ^t**(0.001)^ ^t*(0.030)^	0.000***
Blood Glucose (%)					
Normal	26.33	27.78	27.57	27.62	0.955
High	73.37	72.22	71.36	71.82	0.836
Diabetes home meds. (%)	59.76	66.67	58.35	60.77	0.719
Mean blood LDL (mg/dL)	96.01	99.27	100.30	103.75	0.143
Blood LDL (%)					
Normal	53.11	50.00	50.24	45.86	0.345
High	36.39	44.44	40.45	45.30	0.113
Lipids home meds. (%)	53.55	47.22	48.93	46.96	0.221
Depression home meds. (%)	25.89	11.11	22.20	14.92	0.005**

Hypertension

Blood Pressure Analysis

**Figure 2:**
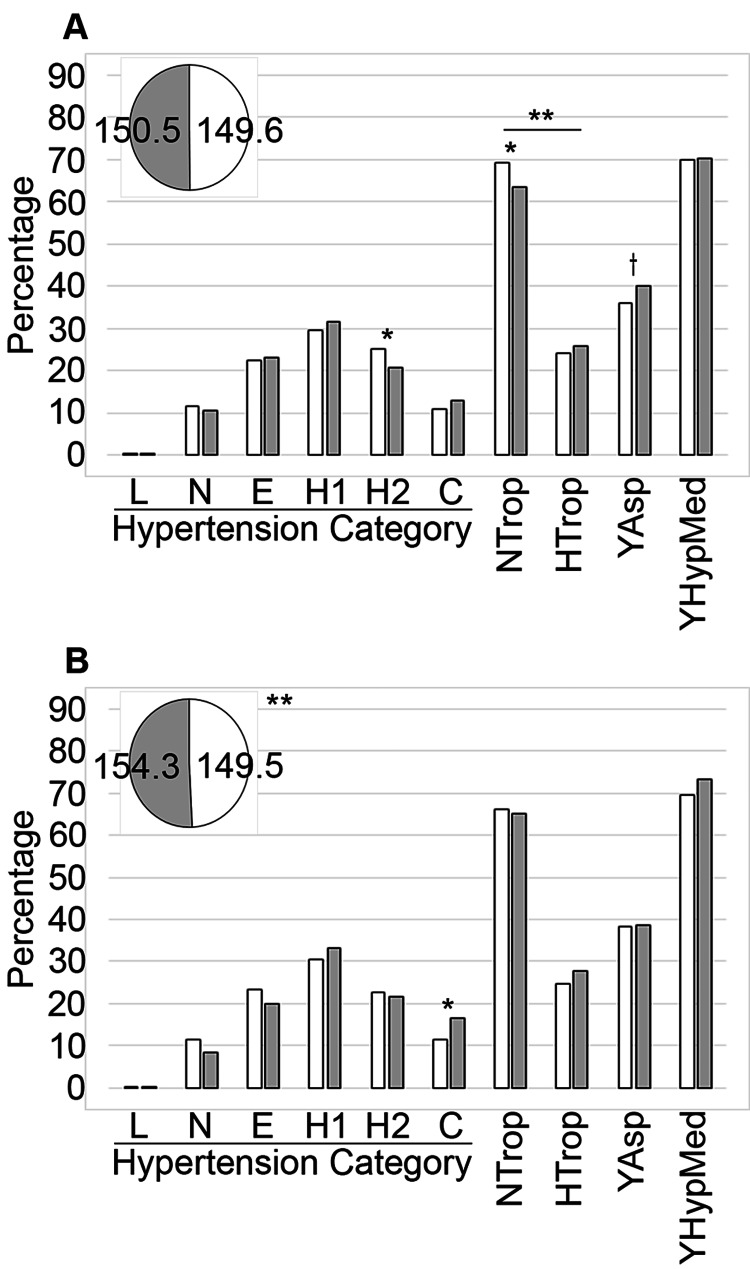
Comparative analysis of hypertension metrics. Data focused on understanding the baseline hypertension status were compared between served and underserved (A) and White and non-White (B) patients. Served patients (n = 712) are represented by the white bars and a pie piece and underserved patients (n = 1,019) by the grey bars and a pie piece in (A). Meanwhile, White patients (n = 1,514) are indicated by the white bars and a pie piece, and non-White patients (n = 217) are shown using the grey bars and pie piece in (B). The mean systolic extraction values are shown in the pie chart inset (mmHg). L: % with low blood pressure; N: % with normal blood pressure; E: % with elevated blood pressure: H1: % in hypertension stage 1; H2: % in hypertension stage 2; C: % in hypertension crisis; NTrop: % with normal troponin 1 levels; HTrop: % with elevated troponin 1 levels; YAsp: % with aspirin as a home medication; YHypMed: % with home medications for hypertension. Symbols indicate significance at the following levels: †p ≤ 0.10, *p ≤ 0.05, **p ≤ 0.01, ***p ≤ 0.001.

We analyzed the first blood pressure reading for each of the 1,731 records by focusing on the value of the systolic extraction (Tables 3-5 and Figure 2). There were three records for which this data was unavailable. We did not observe a notable difference in mean systolic pressure between individuals from served areas (n = 712) or underserved areas (n = 1,019); however, the underserved had a significantly lower frequency in the high stage 2 category (p = 0.038; Table 4 and Figure 2A). None of the served patients were missing these data, but 0.2% of the underserved patients were missing a pressure reading. In contrast, when comparing our hypertension metrics between White (n = 1,511) and non-White (n = 217) patients (Table 5 and Figure 2B), we determined that the non-White patients had a significantly higher systolic extraction (p = 0.009) and that more arrived in crisis (p = 0.029). From the White patient population, 0.13% (n = 2) did not have available blood pressure data from when they arrived in the ED, but the records from the non-White patients were complete. When examining how the combination of both race and service influenced the hypertension metrics (Table 3), we determined that there was a notable variation between these groups, which was particularly pronounced between the underserved White and non-White populations, with the latter having the higher mean systolic extraction pressure. Then, focusing on the systolic extraction categories for each group with an emphasis on the hypertension classifications (high stages 1 and 2 and crisis, Table 3), we determined that there was a significant variation in this data within the high stage 2 category (p = 0.023), but this was not significant enough within the combined high classification.

**Table 4:** Analysis of hypertension-related data for the served and underserved populations. †p ≤ 0.10, *p ≤ 0.05, **p ≤ 0.01, ***p ≤ 0.001

	Served n = 712	Underserved n = 1,019	p-value
Mean systolic pressure (mmHg)	149.6	150.5	0.547
Hypertension categories (%)			0.308
Low	0.42	0.39	
Normal	11.52	10.60	0.548
Elevated	22.47	23.26	0.702
High stage 1	29.49	31.80	0.308
High stage 2	25.14	20.90	0.038*
Crisis	10.96	12.86	0.232
Troponin (%)			0.005**
Normal	69.38	63.69	0.014*
High	24.3	25.80	
Aspirin home meds. (%)	35.96	40.04	0.086†
Hypertension home meds. (%)	69.66	70.36	0.754

**Table 5:** Analysis of hypertension-related data for the White and non-White populations. †p ≤ 0.10, *p ≤ 0.05, **p ≤ 0.01, ***p ≤ 0.001

	White (n = 1,514) populations	Non-White (n = 217) populations	p-value
Mean Systolic Pressure (mmHg)	149.5	154.3	0.009**
Hypertension Categories (%)			0.260
Low	0.40	0.46	
Normal	11.36	8.29	0.177
Elevated	23.38	19.82	0.243
High stage 1	30.52	33.18	0.427
High stage 2	22.79	21.66	0.710
Crisis	11.43	16.59	0.029*
Troponin (%)			0.549
Normal	66.18	64.98	
High	24.83	27.65	
Aspirin home meds. (%)	38.31	38.71	0.910
Hypertension home meds. (%)	69.61	73.27	0.272

Troponin 1 Results

Assessing troponin 1 laboratory data by service level (Table 4 and Figure 2A), there was a significant variation overall (p = 0.005) with more served patients having a normal reading (p = 0.014). Additionally, 6.32% (n = 45) of the served and 10.51% (n = 107) of the underserved did not have these data available. In contrast, we did not observe any differences between White and non-White patients when conducting the troponin 1 analyses. The data were not available for 8.98% (n = 136) of the White patients and 7.37% (n = 16) of the non-White individuals (Table 5 and Figure 2B).

Aspirin and Medications for Hypertension

When we examined the home medications data for hypertension medications or for aspirin specifically, we did not observe any significant differences by service level (Table 4 and Figure 2A) or by racial background (Table 5 and Figure 2B).

Diabetes

Blood Glucose

**Figure 3:**
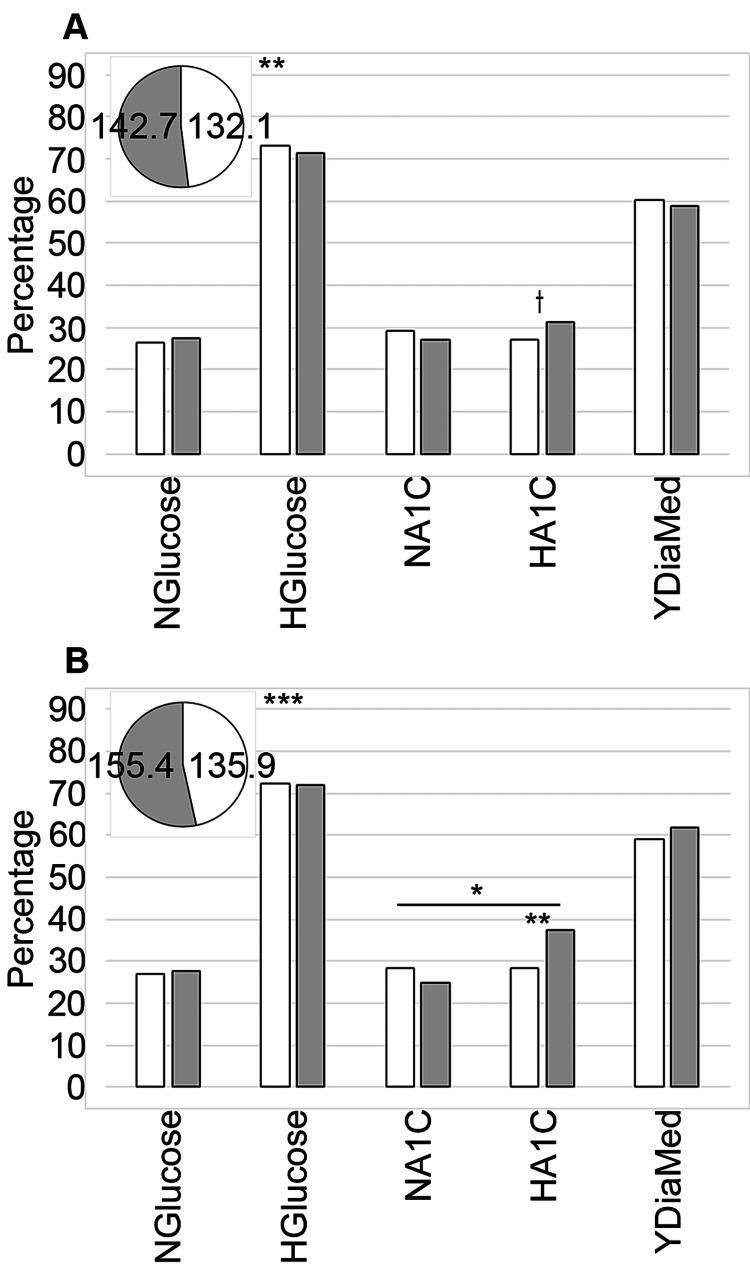
Comparative analysis of diabetes metrics. Data focused on assessing diabetes-related metrics were compared between served and underserved (A) and White and non-White (B) patients. Served patients (n = 712) are represented by the white bars and a pie piece, and underserved patients (n = 1,019) by the grey bars and a pie piece in (A). Meanwhile, White patients (n = 1,514) are indicated by the white bars and a pie piece, and non-White patients (n = 217) are shown using the grey bars and a pie piece in (B). The mean blood glucose values are shown in the pie chart inset (mg/dL). NGlucose: % with normal blood glucose; HGlucose: % with high blood glucose; NA1C: % with normal A1c; HA1C: % with high A1c; YDiaMed: % with home medications for diabetes. Symbols indicate significance at the following levels: †p ≤ 0.10, *p ≤ 0.05, **p ≤ 0.01, ***p ≤ 0.001

Examining the average blood glucose data by service (Table 6 and Figure 3A) indicated that the served had a significantly lower mean assessment compared to the underserved (p = 0.003). However, blood glucose was not sufficiently different between the normal and high categories. The data were not available for 0.28% of the served (n = 2) and 1.08% of the underserved (n = 11). Looking at the mean blood glucose concentrations on arrival by ethnic background (Table 7 and Figure 3B), we observed that the average blood glucose concentration for the White patients was significantly lower than that for the non-White patients (p ≤ 0.001), but that there was no difference between the groups by blood glucose category. The data were not available for 0.79% (n = 12) of the White patients, and 0.46% (n = 1) of the non-White patients. Studying the metrics by service and race (Table 3), we observed interesting mean blood glucose variations between the four groups, which had a particularly significant variation (p ≤ 0.001). The served White patients had a mean blood glucose, which was less than that of the underserved White patients (p = 0.015) and the underserved non-White individuals (p = 0.001). The concentration for the underserved White group was significantly less than that for the underserved non-White patients (p = 0.030). Examining the categorized blood glucose data by service and race did not provide any further significance compared to the service and race data alone.

**Table 6:** Analysis of diabetes-related data for the served and underserved populations. †p ≤ 0.10, *p ≤ 0.05, **p ≤ 0.01, ***p ≤ 0.001

	Served (n = 712)	Underserved (n = 1,019)	p-value
Mean Blood Glucose (mg/dL)	132.1	142.7	0.003**
Blood Glucose (%)			0.136
Normal	26.40	27.58	0.589
High	73.31	71.34	0.368
A1c (%)			0.154
Normal	29.39	26.99	0.280
High	26.97	31.21	0.057†
Diabetes home meds. (%)	60.11	58.78	0.580

**Table 7:** Analysis of diabetes-related data for the White and non-White populations. †p ≤ 0.10, *p ≤ 0.05, **p ≤ 0.01, ***p ≤ 0.001

	White (n = 1,514)	Non-White (n = 217)	p-value
Mean Blood Glucose (mg/dL)	135.9	155.4	0.000***
Blood Glucose (%)			0.857
Normal	27.01	27.65	0.844
High	72.19	71.89	0.926
A1C (%)			0.023*
Normal	28.27	25.18	0.450
High	28.34	37.33	0.007**
Diabetes home meds. (%)	58.98	61.75	0.438

Diabetes Medications

Overall, 59.32% of the patients had medications to treat diabetes included in their home medications list. Analyzing the data we determined that there were no significant differences by service or between White and non-White patients (Tables 3, 6-7, and Figures 3A-3B).

A1c Analysis

Approximately 40% of the patient data was missing the laboratory analysis for A1c. However, since about the same proportion was missing from each of the groups, we decided to include this metric. By service level (Table 6 and Figure 3A), we did not find a major difference between the served and underserved either overall or by A1c category. Additionally, 43.68% of the served did not have this data (n = 311), and it was unavailable for 41.81% (n = 426) of the underserved. By comparison, the A1c data comparing the White and non-White populations were significantly different overall (p = 0.023) and between the two groups for the high classification (p = 0.007). The data were not available for 43.39% (n = 657) of the White patients and 36.87% (n = 80) of the non-White patients.

Lipids

Blood LDL

**Figure 4:**
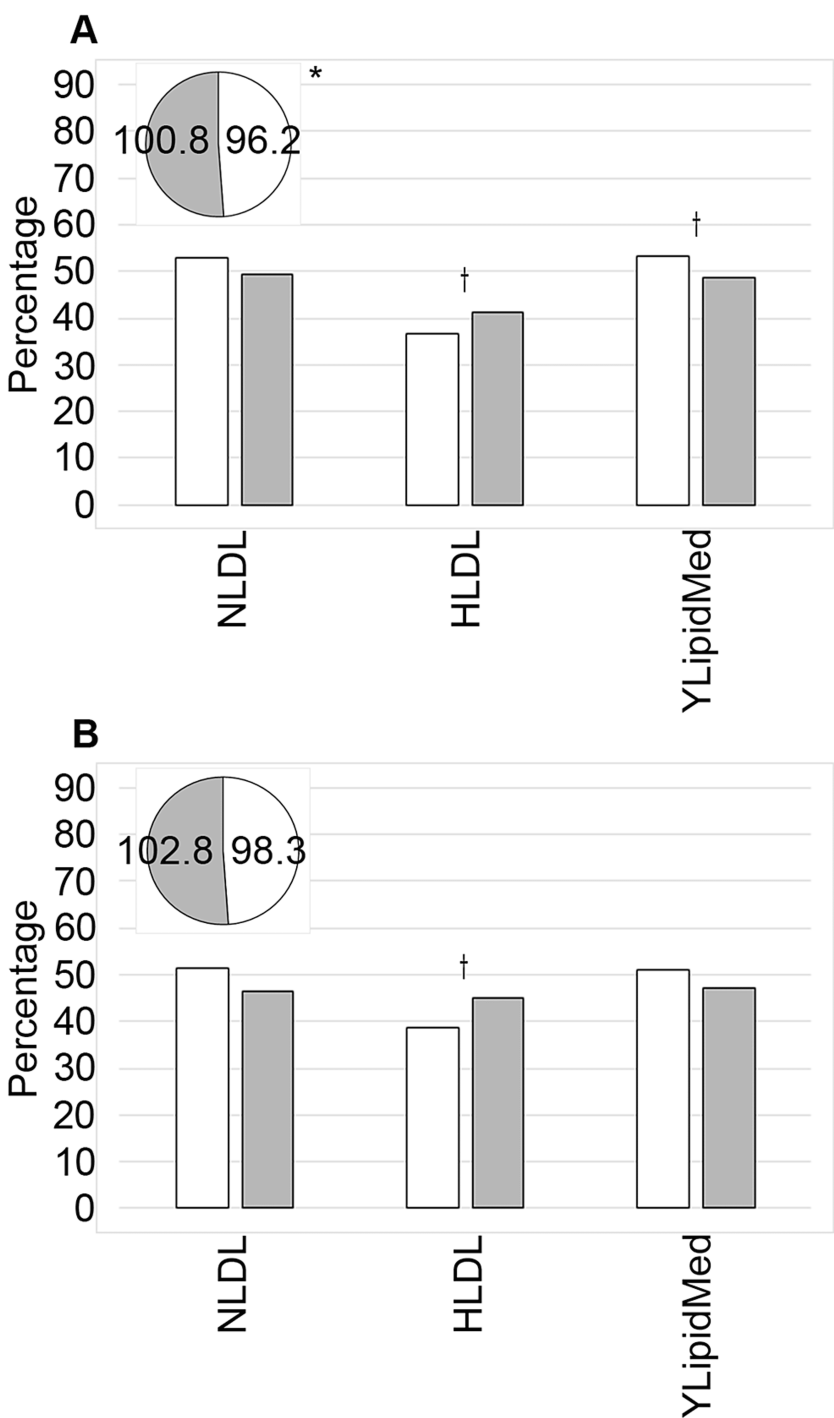
Comparative analysis of lipids metrics. Data focused on assessing blood lipid-related levels were compared between served and underserved (A) and White and non-White (B) patients. Served patients (n = 712) are represented by the white bars and a pie piece, and underserved patients (n = 1,019) by the grey bars and a pie piece in (A). Meanwhile, White patients (n = 1,514) are indicated by the white bars and a pie piece, and non-White patients (n = 217) are shown using the grey bars and a pie piece in (B). The mean blood LDL (low-density lipoprotein) values are shown in the pie chart inset (mg/dL). NLDL: % with normal blood LDL; HLDL: % with high blood LDL; YLipidMed: % with home medications targeting blood lipids. Symbols indicate significance at the following levels: †p ≤ 0.10, *p ≤ 0.05, **p ≤ 0.01, ***p ≤ 0.001

The mean blood LDL concentration for the served (n = 639) was significantly different from the underserved (n = 925; p = 0.041; Table 8 and Figure 4A). There were no notable variations by service for the categorical LDL analysis. The data were not collected on 10.25% of the served (n = 94) and 9.22% of the underserved (n = 73). However, when looking at the numbers by race (Table 9 and Figure 4B), we observed that the average blood LDL concentration for the White population was lower than that for the non-White patients, but the difference was not significant. Similarly, separating the LDL data into high and normal, we did not observe any differences. Additionally, 9.84% of the White patients (n = 149) and 8.29% of the non-White patients (n = 18) did not have these data points. Analyzing the data by both service and race, we determined that this additional breakdown did not increase the variation for the absolute blood LDL analyses or the categorical assessment, but the numbers did follow trends wherein the underserved and non-White patients had the least favorable numbers, the served White patients had the comparatively “better” numbers, and the underserved non-White patients had the “worst” readings (Table 3).

**Table 8:** Analysis of blood lipid-related data for the served and underserved populations. †p ≤ 0.10, *p ≤ 0.05, **p ≤ 0.01, ***p ≤ 0.001

	Served (n = 712)	Underserved (n = 1,019)	p-value
Mean Blood LDL (mg/dL)	96.20	100.8	0.041*
Blood LDL (%)			0.163
Normal	52.95	49.46	0.153
High	36.80	41.32	0.059†
Lipids home meds. (%)		48.58	0.057†

**Table 9:** Analysis of blood lipid-related data for the White and non-White populations. †p ≤ 0.10, *p ≤ 0.05, **p ≤ 0.01, ***p ≤ 0.001

	White (n = 1,514)	Non-White (n = 217)	p-value
Mean Blood LDL (mg/dL)	98.3	102.8	0.172
Blood LDL (%)			0.179
Normal	51.52	46.54	0.170
High	38.64	45.16	0.066†
Lipids home meds. (%)		47.00	0.272

Medications for High Lipids

We determined that 50.49% of patients overall had drugs to treat high lipids included in their home medications list. However, we did not observe any notable differences by service or racial background (Tables 3, 8-9, and Figures 4A-4B).

Depression

We examined the data for evidence of patient depression by checking the home medications list for drugs used for treatment. We observed that, overall, 22.65% of the charts included medications to treat depression. Examining the data by service level, 25% of the served (n = 712) and 20.90% of the underserved patients (n = 1,019) had these medications included in their charts, and this difference was significant (p = 0.038). A greater separation between the data was noted when looking at the charts by race, where we observed that 23.84% of White patients (n = 1,514) and 14.29% of non-White patients (n = 217) had drugs to treat depression included in their home medications list (p = 0.002). We examined this concept further by both race and service (Table 3) and observed a significant variation (p = 0.005), with the non-White patients (served and underserved) having depression medications on their home medications list least frequently. The served non-White patients had the lowest incidence, and the served White patients had the highest.

## Discussion

Even though most stroke patients who arrived in the Crouse Hospital ED were from underserved areas (58.9%), the majority reported having a PCP (92.6%) [42]. Despite this, most patients had hypertension and high blood glucose in spite of the preponderance of the reports including prescriptions to treat these conditions. Non-White patients had the highest blood pressure, blood glucose, and A1c levels and more frequently arrived having had a previous stroke when compared to White patients. Both the underserved and non-White groups had higher NIHSS scores, a greater frequency of ischemic stroke, a lower incidence of TIA, and a greater likelihood of active smoking behavior compared to the served and White patients. In contrast, patient blood LDL levels were largely controlled, and there were no differences in LKW or the use of tPA between the patients. Additionally, White patients were most likely to be prescribed medications to treat depression.

The systolic extraction pressure from the first recorded reading was our standard metric of cardiovascular health because medications are routinely administered in the ED to offset blood pressure issues presented by the patient - we hoped to get a baseline understanding of the patient prior to the administration of these drugs. The majority of patients coming to the ED for stroke had blood pressures that were above normal (88.50% were classified as elevated or higher), with 65.67% presenting with pressures considered “High Stage 1”, “High Stage 2” or “Crisis.” Non-White patients were more frequently categorized as in “Crisis,” with a higher mean systolic pressure when compared to White patients, with the underserved non-White patients having the highest average pressure reading. These findings reflect those made by other groups [44-46]. Notably, when examining the data by race and service, the greatest discrepancy occurred between the underserved White and underserved non-White patients and not between the served White and served non-White categories. This trend was in line with our earlier analysis [42] regarding the social determinants of health wherein we observed that served Black patients were similar to the White patient population and that the differences were most significant for underserved and Black patients - suggesting that access to primary care could work toward addressing health disparities. Elevated troponin 1 and troponin T levels are connected with myocardial injury and are associated with stroke severity; and evidence suggests that stroke patients with increased troponin levels have chronic, instead of acute, underlying heart issues associated with their stroke diagnosis [47,48]. In keeping with the systolic blood pressure results, the troponin 1 analysis indicated that fewer underserved patients had a normal result suggesting that hypertension had a less detrimental effect on the overall cardiac health of the served patients - perhaps due to better access to anti-hypertension medications, and regular PCP visits.

Taking low-dose aspirin as a prophylactic medicine for cardiovascular health was included in our analysis of the home medications lists for hypertension-related drugs because until recently many people used (baby) aspirin daily to support their cardiovascular health. Current advice discourages this use unless upon the advice of a physician (for those with a history of cardiovascular disease or stroke) [49,50]. While most patients did not include aspirin on their home medication list, more charts from the underserved population did. Further analysis of the home medications lists revealed that most patients (70%) did have active prescriptions to address hypertension, which was comparable to the percentage of patients arriving at the ED with pressures classified as “High Stage 1”, “High Stage 2” or “Crisis” (65.67%). While the differences between the patient categories were similar, we observed notable trends when looking at hypertension medications by both race and service. Served non-White patients had hypertension medications on their home medications list least frequently, while the underserved non-White patients were the opposite, having the highest proportion of patients with hypertension medications included on their home medications list. These paradoxical findings provide some insight into the different patient groups, which when combined with the troponin 1 data suggest better overall health for the served groups. The served White population, while older and mostly with Medicare insurance, potentially has better control over their hypertension due to allowances for these prescriptions, when compared to the served non-White groups that are likely composed of younger patients and with possibly fewer provisions offered by their insurance coverage as described in our previous work [42]. The underserved non-White group having the highest incidence of hypertension medications included in their home medication list, still had the greatest systolic extraction pressure, suggesting that their condition is not well managed despite being prescribed the medications to treat hypertension, while others may be dealing with resistant hypertension [51]. Indeed, since 65.57% of the study population was classified as being in “High Stage 1”, “High Stage 2”, or in “Crisis”, and 70.08% of the patients did have medications to treat hypertension included in their home medications list, the results suggest that management of hypertension and/or not following medication regimens, rather than undiagnosed hypertension, is problematic for much of the community of patients regardless of service level or racial background. Indeed, reports indicate that the key to the high percentage of hypertension and stroke is medication non-adherence [52].

Similar to what we observed in our study of hypertension, we determined that the majority of patients arrived with high blood glucose (72.15%), but unlike the hypertension findings, while most patient charts included diabetes medications in their home medication list (59.32%) the proportion was lower than that for patients with high blood glucose, suggesting that a proportion of patients might not realize they have high blood glucose even though nearly all patients stated that they had a PCP [42]. These findings again imply that despite having a PCP, underlying conditions are not well managed, or that patients have not interacted regularly with their PCP. These findings are important in that not only is diabetes a risk factor for stroke (both hemorrhagic and ischemic), but it is also associated with a poorer outcome following a stroke [9,53]. Mean blood glucose levels were significantly higher for both the underserved and the non-White patients, and when combining the data for service and race, blood glucose concentrations were highest among the underserved non-White patients and lowest for those that were served and White. These findings follow the observations of others wherein a greater proportion of the non-White population had high blood glucose and diabetes compared to those that were White [54,55]. Furthermore, the non-White population had a significantly higher average A1c when compared to the White patients, and a significantly greater proportion of patients in the “High” category. This again underlines the propensity for the patients to present to the ED with likely diabetes, or high blood glucose levels as an underlying stroke complication. This analysis was demonstrated to have an important impact on stroke risk, wherein patients with A1c levels above 6.8-7% had a higher risk of stroke within one year [56]. Since hyperglycemia and high A1c are associated with poorer stroke outcomes, moving forward, it is important to make a laboratory assessment of the A1c standard for stroke patients [53,56].

The number of patients with prescribed diabetes medications fell short of what would be expected by the proportion of patients having high blood glucose. The non-White population, and the underserved non-White group in particular, had the highest proportion of patients with medications for diabetes included in their home medications list even though these patients paradoxically also had the highest blood glucose. Again, these findings demonstrate that many patients, and the non-White patients in particular, may not realize that they are diabetic, are not receiving effective or regular primary care to monitor their blood glucose or diabetes - or conversely, are not managing their prescriptions or diagnosis. Similar trends regarding diabetes diagnoses and race were reported by others [54,57-59]. Importantly, as for the hypertension findings, and also in line with what others have observed, we determined the White patients were on average ten years older at the time of their first stroke [42,60-62]. This data is particularly concerning, because the risk of stroke associated with diabetes has been shown to be higher for younger people and that diabetes particularly affects racialized populations [53-55,59].

Fortunately, most patients had never smoked or had quit (84.97% of served, 76.25% of underserved, 81.64% of White, and 67.28% of non-White patients). However, since the underserved and non-White patients were 1.5 times and almost twice as likely to be actively smoking when compared to the served and White patients, respectively, more needs to be done in terms of smoking cessation for these populations. In fact, the combined underserved non-White group had the highest proportion of those actively smoking, with the served non-White patients also being disproportionately affected. The detrimental effects of high smoking rates, particularly among the underserved and racialized communities, are a common concern for many aspects of health, including stroke [63-65]. The importance of smoking cessation to improve the outlook for vascular disease has led to initiatives, such as the one promoted by the Society for Vascular Surgery Vascular Quality Initiative (VQI) [66]. We are also involved in collecting data for this VQI effort and actively provide education to encourage patients who smoke to quit. Looking forward, because of the popularity of vaping, we should take this activity into account as an additional form of nicotine exposure, particularly since vaping is also linked to cardiovascular changes and injury that could pose an increased risk for stroke, and an increased rate of stroke at a younger age [67,68]. While the use of e-cigarettes may pose a lower risk for stroke than conventional smoking behavior, the statistics associated with vaping and stroke are concerning due to the popularity of vaping among young people - both teenagers and adults, women, and racialized individuals [67,68].

The underserved and the non-White patients arrived with fewer instances of TIA, but more frequently for ischemic stroke when compared with the served and White patients, respectively. This trend (racialized groups having higher instances of ischemic stroke) was corroborated by the findings of the Northern Manhattan Study [16]. The lower number of TIA instances among the underserved and non-White patients caused us to question if individuals in these demographics understand that TIA symptoms are stroke-related, or if they choose to forgo treatment for a TIA because their symptoms resolved [69]. Either situation results in a missed opportunity for these patients to connect with healthcare providers and a delay in diagnosing and starting (or reassessing) the treatment of underlying conditions before a more serious stroke [69,70]. As others have reported, the data indicate that non-White patients were significantly more likely to have had a previous stroke or TIA when compared to White patients [71]. Furthermore, the underserved and non-White patients had significantly higher NIHSS numbers and therefore more severe stroke symptoms, when compared with the served and White populations. More specifically, we observed that, while the non-White population had the highest average NIHSS score, the separation of the data points was most significant for the underserved compared to those who were served. We suggest that these findings are related to the possible poorer underlying health of the underserved and racialized populations [9,16,19,20].

The time between the onset of symptoms and arrival to the ED for stroke is a critical metric in deciding a course of treatment, so we conducted a further analysis of the LKW data to determine how the findings may influence the stroke outcomes of the patients. In contrast to what others have observed, and despite the variation in stroke diagnoses between the patients, significant differences by service (despite the longer travel time for many of the rural/underserved patients) or race when it came to LKW were not observed [72,73]. To be considered for thrombolytic treatment, patients must have had an ischemic stroke and be less than 4.5 hours from their LKW. Imaging results and the NIHSS score are other aspects that are also considered prior to tPA administration. Applying the LKW data, as well as the type of stroke, as two of the medical qualifications for tPA treatment, we determined that there was no significant difference between the proportion of patients that received tPA when comparing the data by service or race. These findings provide an important contrast between our hospital and others who reported discrepancies in tPA treatment by race due to declination of thrombolytic treatment or later arrival times causing treatment disparities for racialized patients [74-77].

In contrast to both the hypertension and diabetes observations, most patients did not have high lipids - 39.46% of patients arrived with a high blood LDL and about half of the patients (50.49%) had lipid-lowering drugs included on their home medications list - exceeding the proportion of patients with high blood LDL. However, the underserved had significantly higher blood LDL results when compared to the served population, while the proportion of those with lipid-lowering prescriptions was higher for those who were White and the served population and the lowest for individuals who were underserved and non-White population. Others observed that racialized individuals can have higher lipid levels and atherosclerosis than those who are White and suggest that this trend contributes to the higher risk of stroke among the affected populations [78,79]. The use of statins and lipid-lowering drugs reportedly has a positive impact on cardiovascular health in ways that are just now being understood [80-83]. Exactly how these medications benefit and how high blood lipid levels relate to stroke is still an active area of investigation [79,84,85]. Regardless of the differences between groups, the observations suggest that blood lipid levels are better maintained than hypertension and diabetes, even among non-White patients.

It is not uncommon for people who suffer from depression to have a stroke [86-88]. A recent study determined that individuals with prolonged depression trajectories had an increased risk of stroke and that a diagnosis of diabetes is associated with an increased rate of depression and cardiovascular disease [29,89]. More than a fifth of the patient records included medications to treat depression, and those patients from served areas were more likely to have these medications on their home medications list compared to underserved patients. Furthermore, White patients had medications to treat depression on their home medications list at a rate that was 10% higher than that of non-White patients, with served White patients having the highest propensity and underserved non-White patients, the lowest. The findings correlate with published work indicating that White individuals are treated for depression more frequently than racialized individuals [90,91]. Unfortunately, depression can go undiagnosed, particularly among those with lower socioeconomic status and non-White groups, who might also be less likely to seek help when they are suffering from depression [92]. Others observed that depression occurs at a higher rate in the non-White population, and that (once socioeconomic status is accounted for) the White population may be less resilient to the onset of depression [93,94]. Regardless of the underlying complexities associated with depression, race, and socioeconomic status, Soh et al. determined that those with declining depressive incidents had the same stroke risk as others without depression, suggesting that there is a long-term benefit for stroke patients by making strides to support their mental health while addressing underlying cardiovascular issues [89].

Limitations

Since this is a retrospective study, we did not have contact with the patients to question them about their knowledge of possible underlying conditions or determine how frequently they visited their PCP. Our analysis was also limited to the ED record and did not consider other outlying risk factors for stroke in the patient history (e.g., genetics, human immunodeficiency virus infection, or history of preeclampsia), or those aspects that may directly affect the risk for certain types of stroke such as cardioembolic stroke, which can be influenced by hypercoagulable disorders, such as cancer or history of cancer, autoimmune or connective tissue disorders or the presence of an irregular heart rate such as atrial fibrillation [9]. It was also not possible for us to question former smokers about how long ago they stopped smoking, so this information is not factored into the analysis. The data examining racial background would be more robust with a larger non-White patient cohort. Our A1c and troponin 1 results were limited because these analyses were not included in many of the laboratory results. We also acknowledge that, since the data are focused on one hospital, they may not reflect the findings of other centers in the area. Other stroke data on those in the community who suffered a stroke and did not seek medical care, or succumbed to their stroke without the opportunity to come to the ED could not be included in the analysis [95]. The data also did not account for how the severity of symptoms affected how long patients took before seeking medical care [96]. However, despite these limitations, we believe that our findings are sufficiently robust to provide key insights into the underlying health of the patient population to develop strategies to improve community stroke outcomes.

Action plan

The main action item from our previous analysis involving the social determinants of health in the study population was to provide a bridge between the patient-centered focus of the hospital setting and continued care in the form of clinical follow-up visits by having the clinic nurse meet with stroke patients prior to discharge to assess the individual needs of each patient and stress the importance of connecting with their PCP [42]. To address the findings from this report and to continue a patient-centered focus, we believe that it is also important to determine the effectiveness of stroke education efforts and then take action to improve where we might be falling short. Follow-up visits already stress the importance of smoking cessation and scheduling appointments with providers including specialists and PCPs. Even so, we believe that it is key to assess patients for their understanding of the role of their PCP and their appreciation of the importance of addressing underlying conditions that might have contributed to their stroke. Patients may need help learning the steps needed to control hypertension and diabetes, in particular, and providers should cooperate to work toward an effective plan of action to help patients negotiate their continued care [2,6,7,97].

We also believe that there is a disconnect for many stroke patients prior to their stroke in accepting how their own state of health may increase the risk of stroke and an underappreciation of the connection between underlying health conditions and certain behaviors such as smoking [8,98]. This misconception contributes to the neglect of primary care and continued smoking. These effects can be further exacerbated by limitations in health insurance and less access to primary care that may be common in underserved and racialized communities. Indeed, the findings demonstrate that the Black population had a higher proportion of individuals with Medicaid insurance compared to White patients. While many of the medical aspects assessed here do not show major changes by race or service, the data indicate that Black patients came to the ED for stroke about 10 years earlier than White patients, according to the findings of our earlier study [42]. This 10-year difference can affect the level of care as Medicare currently has more provisions for stroke and the associated risk factors for the older population when compared to other forms of insurance such as Medicaid [42,99,100]. Given these considerations, we believe that an effective strategy must strive to consider the patient situation and understanding of stroke to have a meaningful impact.

To begin, we plan to assess the patient's perspective on stroke risk factors and the role of their PCP in the form of an anonymous survey in the clinic at the follow-up visit and then again after one year to determine if there is a shift in outlook when they are further out from their stroke. The use of surveys to track the success of stroke education has been demonstrated as effective by others [35]. The questions for our survey (Appendix Table 10) will be provided to patients either using a tablet or iPad with large font and touchable radio buttons, so patients can easily make the Likert scale selections or by mailing it to the patients and including a self-addressed stamped envelope. The survey questions are designed so that patients can reflect on their perspectives before having their stroke and compare them to those after their stroke. Along with the survey questions, we will collect basic demographic information, such as age, sex, racial identity, zip code, and level of education. We will retrospectively review the findings and compare the responses between the served and underserved and White and non-White patients. This will allow us to assess the success of our stroke education and to determine if the knowledge was gained equitably between the patient categories. The survey will help us refine our messaging to increase patient understanding of risk factors and motivate patients to seek assistance in addressing the issues that pertain to them.

## Conclusions

Even though most patients reported having a PCP, the majority of the stroke incidents were associated with high blood pressure and high blood glucose, despite the prevalence of prescriptions to treat these and other underlying conditions. However, hypertension and high blood glucose more significantly affected the underserved and non-White patients. Notably, the served non-White patients had an average systolic extraction that was comparable to that of the served White patients, indicating that served patients do have an advantage over the underserved, regardless of racial background, and that residing in a served area can have an important impact on the health of non-White patients. These results imply that maintenance of cardiovascular health, rather than undiagnosed hypertension, is an issue that should be addressed in the patient population. When it came to smoking, the underserved and non-White patients, with the underserved non-White patients in particular, had a significantly higher tendency to exhibit smoking behavior. Overall, the observations stress the importance of having consistent primary care to keep track of underlying conditions contributing to stroke. This is particularly evident in light of the disparities faced by underserved and non-White patients in reference to diabetes and smoking as critical features affecting stroke. This disparity is even more striking given that most ischemic strokes and high NIHSS scores were associated with underserved and non-White patients and that more non-White patients arrived having already had a previous stroke. Understanding the risk of stroke from smoking and uncontrolled medical conditions are key aspects of patient education that can be provided by PCPs, as well as by community outreach and specialist provider messaging. Given that the rate of stroke in the United States is predicted to escalate and that young people will be increasingly affected, it is important to develop approaches to address these issues. It is our hope that focused patient education as assessed by in-clinic patient feedback will help us, as well as providers at other institutions, improve post-stroke outcomes and continue to make inroads toward reducing the burden of stroke in the community.

## Appendices

**Table 10:** Stroke education survey questions This anonymous survey will record patient perspectives on primary care and underlying health risk factors shortly after their stroke and after one year. The questions will be presented as a large-font digital document with radio buttons for the Likert scale ratings. The survey will be provided to patients using a tablet or iPad in the clinic at the end of their follow-up appointment. Alternatively, the survey will be mailed to patients along with a self-addressed stamped envelope. Other demographic information such as age, race, sex, zip code, and education level will also be collected.

Thank you for taking a few minutes to answer some questions about your perspectives before and after your stroke. Your answers will help our team learn how we can best support you and other stroke patients. Please select the best answer from those provided. The responses are anonymous and your participation is voluntary.	
1. Before your stroke, how well did you understand the risk for stroke associated with diabetes, hypertension and smoking?	
1	I had no idea that diabetes, hypertension and smoking increased the risk for stroke.
2	I heard something about diabetes, hypertension and smoking increasing the risk for stroke, but did not appreciate the connection.
3	I knew that diabetes, hypertension and smoking increased the risk for stroke, but needed help addressing the factors that related to me.
4	I knew that diabetes, hypertension and smoking increased the risk for stroke and did address the factors that related to me.
5	I knew that diabetes, hypertension and smoking increased the risk for stroke, but none of these factors applied to me.
2. How long before your stroke was your last visit with your primary care provider?	
1	I do not have a primary care provider.
2	I last visited my primary care provider 10 or more years before my stroke.
3	I last visited my primary care provider between 5 and 10 years before my stroke
4	I last visited my primary care provider between 1 and 5 years before my stroke.
5	I last visited my primary care provider within the year before my stroke.
3. Before your stroke, how confident were you in your ability to manage your to manage your diabetes, hypertension and smoking?	
1	I was not at all confident that I could manage my risk factors before my stroke.
2	I was trying, but I was having trouble managing my risk factors before my stroke.
3	I was somewhat confident that I was managing my risk factors before my stroke
4	I was confident that I was managing my risk factors before my stroke.
5	None of these risk factors applied to me before my stroke.
4. Before your stroke, how important was it to you to manage your diabetes, hypertension and smoking?	
1	I did not consider these risk factors as important before my stroke
2	I did consider it was important to manage my risk factors, but I was too busy with other things before my stroke, so I did not address them.
3	I thought it was important to manage my risk factors, but I did not consistently make an effort to do so.
4	I thought managing my risk factors was important before my stroke and did my best to do so.
5	None of these risk factors applied to me before my stroke.
5. How has your knowledge of the risk of stroke associated with diabetes, hypertension and smoking changed since having your stroke?	
1	My knowledge of these risk factors has not increased since having my stroke.
2	My knowledge of these risk factors has increased a little, but I feel overwhelmed by the information.
3	My knowledge of these risk factors has increased somewhat, but I would like more information.
4	My knowledge of these risk factors has increased a lot, but I would like more information.
5	My knowledge of these risk factors has increased a lot, and I feel confident in what I have learned.
6. How satisfied are you with the resources you received during your post stroke care to help manage your diabetes, hypertension and smoking?	
1	Not at all satisfied.
2	Somewhat unsatisfied.
3	None of these risk factors apply to me, or neither satisfied or unsatisfied.
4	Satisfied.
5	Very satisfied.
7. How likely are you to follow the advice of your primary care provider to manage your diabetes, hypertension and smoking after your stroke?	
1	I do not have a primary care provider to help me manage my risk factors.
2	It is unlikely that I will follow the advice of my primary care provider to help me manage my risk factors.
3	I do not have a primary care provider but would like help finding one to help me manage my risk factors.
4	It is likely that I will follow the advice of my primary care provider to help me manage my risk factors.
5	None of these risk factors apply to me.
8. How frequently do you intend to visit your primary care provider in the future?	
1	I do not have a primary care provider and do not intend to visit one.
2	I intend to visit my primary care provider only when I am not feeling well.
3	I intend to find a primary care provider to begin regular visit with one.
4	I intend to visit my primary care provider at least annually to monitor my health and manage any risk factors.
5	I intend to visit my primary care provider at least annually, and more often as needed to manage my risk factors.
